# Building consensus on interactions between population health researchers and the food industry: Two-stage, online, international Delphi study and stakeholder survey

**DOI:** 10.1371/journal.pone.0221250

**Published:** 2019-08-22

**Authors:** Katherine Cullerton, Jean Adams, Oliver Francis, Nita Forouhi, Martin White

**Affiliations:** 1 MRC Epidemiology Unit & Centre for Diet and Activity Research, Institute of Metabolic Sciences, University of Cambridge, Cambridge, United Kingdom; 2 School of Public Health, University of Queensland, Herston, Australia; Shahjalal University of Science and Technology, BANGLADESH

## Abstract

Key to scientific integrity is ensuring that research findings are considered credible by scientific peers, practitioners, policymakers and the public. Industry sponsorship of nutritional research can result in bias and raises significant professional, public and media concern. Yet, there is no international consensus on how to prevent or manage conflicts of interest for researchers considering engaging with the food industry. This study aimed to determine internationally agreed principles to guide interactions between population health researchers and the food industry to prevent or manage conflicts of interest. We used a two-stage, online Delphi study for researchers (n = 100 in 28 countries), and an online survey for stakeholders (n = 84 in 26 countries). Levels of agreement were sought with 56 principles derived from a systematic review. Respondent comments were analysed using qualitative content analysis. High levels of agreement on principles were achieved for both groups (researchers 68%; stakeholders 65%). Highest levels of agreement were with principles concerning research methods and governance. More contentious were principles that required values-based decision-making, such as determining which elements of the commercial sector are acceptable to interact with. These results provide the basis for developing internationally-agreed guidelines for population health researchers governing interactions with the food industry.

## Introduction

The commercial food sector is widely recognised as an important driver of consumer food environments and population diets.[1, 2] With dietary risk factors responsible for a substantial proportion of the burden of non-communicable diseases (NCDs),[3] momentum is growing to identify ways to reduce the adverse influence of the food industry by changing industry behaviour, leading to healthier consumer food on offer.[4, 5] A number of agencies have identified the challenges for policymakers of working with the food industry to stimulate such change.[6, 7] Identifying interventions involving the food industry that can achieve change in population diets is also a significant challenge for the research community.

The debate concerning whether researchers who study food choices, population diets and nutrition (referred to hereafter as population health researchers) should interact with the food industry has led to calls for clear guidance for researchers.[4, 8–10] For such guidance to be acceptable and useful, consensus needs to be reached on the conditions under which it may be acceptable to interact with the food industry. Many population health researchers highlight the negative consequences of interacting directly with the food industry, including: immediate and future reputational risk for researchers; misdirection of the research agenda; unconscious bias in the interpretation and reporting of research findings; and decreased trust in research outputs.[8–13] Those in favour of interacting with the food industry argue that, with scarce government funding for research in many countries and the potential for positively influencing practices within the food industry, ways to interact without risk of reputational or other consequences need to be found.[4, 10, 14–16]

A key challenge for researchers considering interacting with the food industry is the risk of conflicts of interest. A conflict of interest is defined as “a set of circumstances that creates a risk that professional judgement or actions regarding a primary interest will be unduly influenced by a secondary interest”.[17] Preventing and managing actual or perceived conflicts of interest is important for three reasons. Firstly, it will help to protect the integrity of researchers’ professional judgements.[17] Secondly, it will help to minimise conditions that would cause a reasonable person (colleague or citizen) to believe that professional judgement has been improperly influenced, whether or not it has.[18] Finally, interacting with the food industry requires researchers to exercise judgement in contexts with which they may be unfamiliar (e.g. the commercial context) and that may be fraught with uncertainty for them.

To provide a foundation for this study, we undertook a systematic scoping review to identify the range of documented principles to prevent and manage conflicts of interest between population health researchers and the food industry.[19] The review found consistency among authors concerning standards of research governance, transparency and publication, but there was less agreement on how general principles should be applied in relation to assessing the appropriateness of an industry partner and the type of interaction with researchers. This is perhaps unsurprising as a decision to interact with the food industry involves values-based decision-making and, as a result, there is a wide range of opinions. What is seen as crucial for credibility and integrity by one person may appear of marginal importance to others.

In order to build further consensus on these issues, in this study we sought the views of the population health research community and research stakeholders (e.g. funders, policy officers and journals) internationally.

## Methods

### Study A: International Delphi study

#### Study design

To survey researcher opinion and build consensus, we used a two stage, online international Delphi study. The Delphi method is widely used for consensus building particularly where there is contradictory or insufficient existing information–as was the case here.[20] It typically uses a series of questionnaires delivered using multiple iterations to collect expert opinion and feedback from selected participants.[21] The iterative nature of the Delphi method helps to quantify support and agreement, exposes participants to alternate view points; and allows them time to reconsider their responses and the extent to which they share the views of others.[21] All study procedures were approved by the University of Cambridge School of Humanities and Social Sciences Research Ethics Committee.

#### Participant recruitment

A multi-pronged, purposive approach to recruitment was adopted with the aim of recruiting the largest and most geographically diverse possible sample of population health researchers working on diet or nutrition. The process began with invitations to UK based dietary public health researchers from an existing internal database from the author’s workplace plus members of the World Public Health Nutrition Association (accessed via www.wphna.or) who identified as researchers. Chairs/Presidents of national public health nutrition organisations around the world were identified via Google searching and sent invitations to participate, with a particular emphasis on low and middle-income countries. The Chairs/Presidents were asked to forward the invitation on to relevant members within their association. Editorial members of public health nutrition journals who identified as researchers were also sent invitations to participate.

The inclusion criteria (checked at the start of the survey) were researchers: (a) currently involved in population health research; (b) not employed by the food industry (although receiving grant money from the food industry currently or previously was acceptable); (c) conducting research that did not primarily focus on agriculture or food technology; (d) with access to a computer; (e) who understood English; and (f) were able to provide informed consent to the study procedures. Once they had agreed to participate, written informed consent was obtained from each participant.

#### Survey development

Typically a Delphi survey commences with open-ended questions designed to generate ideas that inform the development of principles/statements.[22] As we had previously identified 56 principles proposed to govern relationships between researchers and the food industry in our systematic scoping review of peer-reviewed and grey literature [19] we used these principles to form the basis of the Delphi survey. The principles were individually listed under headings representing the five themes identified in the scoping review: funding, conducting a risk assessment, research governance, transparency and publication. Participants were asked to indicate their level of agreement with each statement on a 5-point Likert scale (1 = strongly agree, 2 = agree, 3 = neither agree or disagree, 4 = disagree, 5 = strongly disagree). A free text box beside each statement and at the end of the survey gave participants the opportunity to make further comments. We encouraged participants to indicate, if they did not fully agree with a statement, in what ways they felt each statement would need to be altered in order for them to agree with it.

Participants were asked about their place of work, the focus of their research, years employed in research, main country of practice and whether they had previously interacted with the food industry. Before distribution to participants, the Delphi survey was pilot tested with ten key personnel who work in population health research in a range of countries to ensure that the items and the wording were relevant and appropriate for an international audience.

#### Data collection and analysis

Participants who expressed an interest in participating were sent the online survey and a unique login ID via email and responses were anonymously linked to the participant’s ID number. Reminder emails were sent each week and any participant not replying after three weeks was considered a non-responder.

As the aim of this study was to build consensus, we summarised level of agreement (‘agree’ and ‘strongly agree’) with each principle as a percentage of all participants. Consensus is often set at 75% agreement in Delphi studies.[22, 23] However, as this issue is highly emotive,[10, 24, 25] we set consensus at 80% agreement among participants. Responses to open-ended questions seeking feedback on statements were analysed using content analysis to assist in the refinement for round 2 of the Delphi survey.[20] Statements that were unclear in round 1 of the Delphi study were modified to improve clarity in round 2; in addition, six new statements were derived from participant feedback and introduced in round 2.

#### Round 2 revisions

During round 2 of the Delphi study, all round 1 participants were emailed to ask them to log in to the online system for a second time. They were presented with the key principles that achieved consensus in round 1 and the percentage agreement was provided beside each statement. They were then guided through the same process as in round 1 for those statements that did not reach consensus, together with the new statements. Each participant could see their previous ratings and comments from round 1 for each statement that did not reach consensus and these could be changed for round 2. Reminders were sent as in round 1. Consensus was again defined as at least 80% agreement. Free comments provided by participants were again analysed using content analysis. Illustrative quotes representing the diverse viewpoints on issues that had lower levels of agreement were identified, and anonymised for presentation. Quotes are accompanied by the research focus of the researcher plus their country of work.

### Study B: Stakeholder survey

#### Study design

To survey the opinions of research stakeholders, we used a single round online survey based on the Delphi survey used in Study A. We used a survey rather than a Delphi study, since we did not aim to build consensus among research stakeholders, but instead seek their opinions to inform consensus building among researchers. All study procedures were approved by the University of Cambridge School of Humanities and Social Sciences Research Ethics Committee.

#### Participant recruitment

A similar approach to recruitment was adopted for the stakeholder survey with the aim of recruiting the largest and most geographically diverse sample of research stakeholders possible. The process began with invitations to UK based policy officers, policy advocates, research funders and journalists with an interest in diet and population health from an existing in-house database that included contacts of authors, together with selected members of the World Public Health Nutrition Association who identified themselves as research stakeholders. Invitations were also sent to the CEOs of national public health nutrition charitable and funding organisations as well as government nutrition departments around the world, with a particular emphasis on low and middle income countries, who were asked to forward the invitation on to relevant members.

The inclusion criteria (checked at the start of the survey) were participants had to have a current work role that involved any of the following: developing new or reviewing existing government policy related to food, diet and/or nutrition and public health; managing research funding applications related to food, diet and/or nutrition and public health; attempting to influence local, regional or national government policy related to food, diet and/or nutrition and public health. Participants also needed to have access to a computer, understand English, and be able to provide informed consent to the study procedures. For those who agreed to participate, written informed consent was obtained from each participant.

#### Survey development

The Delphi survey used in round 2 in Study A, plus the statements which reached consensus in round 1 were used as the basis for the stakeholder survey. As a number of Delphi participants identified the substantial time burden of completing the Delphi study, we aimed to reduce the number of items in the stakeholder survey where possible. As a result, two statements that received very high levels of agreement in the Delphi study were removed. Consistent with the Delphi study, in the stakeholder survey the level of agreement for each statement was measured on a 5-point Likert scale ranging from strongly disagree to strongly agree and a free text box was available beside each statement and at the end of the form. Participant characteristics were also collected, as in the Delphi study.

#### Survey data collection and analysis

Data collection and analysis followed the same process as in Study A. As we were not seeking consensus in the Survey, we measured levels of agreement for each statement. Status of ‘agreement’ was assigned when at least 80% of participants agreed or strongly agreed with a statement.

## Results

### Response rates and participant characteristics

#### Study A: Delphi study of researchers, round 1

One hundred and forty individuals expressed an interest in participating in the Delphi survey. In round 1, 104 completed responses were received. Four participants were ineligible to participate leaving 100 eligible participants. Participants represented 28 countries: 59% from high-income countries, 37% from middle-income countries, and 3% from low-income countries. The main employer was a higher education institution (82% of participants), while 7% were employed by non-government organisations. All participants were diet, food or nutrition researchers, with 70% being highly experienced having worked in the field for more than 10 years. The most common research focuses of participants were: the development, analysis or evaluation of food and nutrition policy (53%, n = 53); the development or evaluation of behavioural change interventions to improve diet/nutritional status of the population (52%, n = 52); and nutritional epidemiology (41%, n = 41). Participants were able to tick more than one option and so percentages do not sum to 100.

#### Study B: Stakeholder survey

For the stakeholder survey, 104 individuals expressed an interest in participating and were sent the survey, and 84 individuals completed the survey. Participants represented 26 different countries (high-income 58%, medium-income 35%, low-income 8%). Most participants were either government policy officers (n = 39, 46%) or from the charitable sector (n = 34, 40%). The majority of participants had worked in the sector for ten or more years (n = 55, 65%).

A sizeable proportion of participants from both groups reported having interacted with the food industry within the previous five years (Table 1).

**Table 1:** Participants’ level of interaction with the food industry within the past 5 years and more than 5 years ago.

	Number (%) engaging	
	Within the last 5 years	>5 years ago
**Type of interaction with the food industry: Researchers**		
Received direct funding	16 (16)	16 (16)
Received in-kind funding	29 (29)	8 (8)
Engaged in formal dialogue	43 (43)	17 (17)
**Type of interaction with the food industry: Stakeholder’s organisation**		
Received direct funding	13 (15%)	3 (4%)
Received in-kind funding	15 (18%)	3 (4%)
Engaged in formal dialogue	53 (63%)	2 (2%)

#### Consensus—Researcher Delphi survey

Consensus (80% agreement or higher) was reached for 28 of the 56 statements presented in round 1 of the Delphi survey (Table 2—responses are colour coded depending on level of agreement). The highest levels of agreement were seen for those statements that addressed ‘ensuring the public is at the centre of the agreement’, ‘management of conflict of interest’, ‘consequences’, ‘transparency’ and ‘publication’. Modifications resulting from participant feedback were made to ten remaining statements, three statements were removed and six new statements were developed for round 2. These new statements either provided greater clarity on an issue or incorporated new concepts derived from feedback. This resulted in 28 statements being included in round 2 of the Delphi survey.

**Table 2:** Results from the Delphi study of population health researchers and the survey to stakeholders.

Statement	Round 1 Delphi Researchers (N = 100), Number (%) agreeing with statement	Round 2 Delphi Researchers (N = 92), Number (%) agreeing with statement	Stakeholder Survey (N = 84) Number (%) agreeing with statement
**1. Funding**			
1.1 A pool of funding from the food industry which is independently administered by a publically accountable third party should be created	74 (74)	79 (86)	53 (63)
1.2 A system where industry provides funding to research institutions, not individual researchers or research units, should be created	34 (34)	29 (32)	25 (30)
1.3 Researchers should not accept funds from the food industry	47 (47)	40 (43)	59 (70)
1.4 Researchers should not accept funds from processed food companies	53 (53)	51 (55)	67 (80)
1.5 Researchers should not accept funds from any commercial organisation	24 (24)	21 (23)	25 (30)
**For those who accept funding from the food industry**			
1.6 Researchers should have no commercial interest in the product being researched	91 (91)	Not included in Round 2	80 (95)
1.7 Funding from industry should reflect the full cost of the research (e.g. using standard academic costing) and not more than this amount	70 (70)	74 (80)	55 (65)
1.8 Industry funding should be non-designated	70 (70)	65 (71)	63 (75)
1.9 There should be no involvement of a food industry funder in any aspect of a research project	70 (70)	67 (73)	70 (83)
**2. Undertake thorough Risk Assessment**			
2.1 Have a clearly identified system to identify and assess interests of potential partners	95 (95)	Not included in Round 2	79 (94)
2.2 A partnership should only be initiated if it will help advance the public health goal	74 (74)	73 (79)	53 (63)
2.3 Only enlist partners who are committed to long term funding and engagement	35 (35)	32 (35)	19 (23)
2.4 Only enlist partners who are committed to sharing of research data arising from the research project	77 (77)	79 (86)	74 (88)
2.5 Only enlist partners who operate in an ethical manner and uphold the human rights of women, men and children	89 (89)	Not included in Round 2	75 (89)
2.6 Ensure the organisational values and overarching goals of the partners are compatible	81 (81)	Not included in Round 2	60 (71)
2.7 Ensure all partners have shared objectives and a shared approach to the research question and activities	77 (77)	74 (80)	61 (73)
2.8 Avoid companies whose objectives and/or goals are related to the increased production, supply or demand of ‘unhealthy food’ products and/or to the promotion of unhealthy and unsustainable ways of eating and producing food	76 (76)	69 (75)	69 (82)
2.9 Assess whether the partnership could undermine the integrity or trustworthiness of my institution	98 (98)	Not included in Round 2	83 (98)
**Risk Assessment of type of engagement**			
2.10 Consider whether the proposed engagement would be acceptable across institutions and national borders’	68 (68)	72 (78)	71 (85)
2.11 Be guided by generic international protocols and frameworks (e.g. World Health Organisation) on appropriate types of engagement	91 (91)	Not included in Round 2	72 (85)
**Ensure public benefit is at centre of agreement**			
2.12 Consider whether the partnership provides maximum benefit to society	89 (89)	Not included in Round 2	68 (81)
2.13 Consider what the public would think about this arrangement	84 (84)	Not included in Round 2	68 (81)
**Consider possibility of reputational damage and loss of trust**			
2.14 Consider what my colleagues would think about this arrangement	71 (71)	70 (76)	64 (76)
2.15 Decline to give a presentation at events sponsored by the food industry	Not included in Round 1	42 (46)	24 (29)
2.16 Decline funding (e.g. travel costs or honorarium) from the food industry to present research findings at an event	Not included in Round 1	58 (63)	47 (56)
2.17 Do not ‘ghost-write’ publications for the private sector	92 (92)	Not included in Round 2	74 (88)
2.18 Do not accept gifts or hospitality if it compromises or appears to compromise objectivity	97 (97)	Not included in Round 2	84 (100)
2.19 Do not participate in undisclosed paid authorship arrangements in industry-sponsored publications or presentations	97 (97)	Not included in Round 2	82 (98)
2.20 Do not allow the commercial partner to co-brand (e.g. use their logo) on the research project or related material	77 (77)	73 (79)	62 (74)
**3. Research governance**			
3.1 Clearly state & agree goals, objectives, roles and responsibilities and accountability before work commences	97 (97)	Not included in Round 2	83 (99)
3.2 Plan research so it is designed objectively and is scientifically sound in its approach	98 (98)	Not included in Round 2	Removed for stakeholders
3.3 Establish up-front control and ownership of the data by the researcher/s irrespective of the funding source	Not included in Round 1	86 (93)	83 (99)
3. 4 Provide open access to anonymised data and analyses once results are published	Not included in Round 1	81 (88)	75 (89)
3.5 Data analysis should be done by statisticians independent of the researcher/s who designed and conducted the study	52 (52)	43 (47)	51 (61)
3.6 Undertake random audits of data provided by food companies for research projects	76 (76)	76 (83)	71 (85)
3.7 Secure oversight of the research by a non-conflicted third party	74 (74)	73 (79)	68 (81)
3.8 Require all trials or other studies in dietary public health to be registered at time of initiation of the study	89 (89)	Not included in Round 2	72 (86)
**Ensure partners have equal power**			
3.9 If the food industry is supporting research by providing direct funding or data, ensure they do not have undue influence over research by having a diversity of partners on project steering committees (e.g. NGOs, consumers).	Not included in Round 1	76 (83)	76 (90)
3.10 The research institution must be able to independently criticize a commercial-sector entity for issues unrelated to the partnership	96 (96)	Not included in Round 2	80 (95)
**Ensure public benefit is at centre of agreement**			
3.11 Engage independent members of the public in the process of defining research problems and subjecting research projects to ongoing critical scrutiny	71 (71)	69 (75)	58 (69)
**Management of conflict(s) of interest**			
3.12 Have a clearly identified system to identify, assess and manage the interests of all stakeholders	97 (97)	Not included in Round 2	82 (98)
3.13 Recuse stakeholders from committee (or similar) decision making where there may be an actual or perceived conflict	88 (88)	Not included in Round 2	76 (90)
3.14 Continuously monitor for conflicts of interest	96 (96)	Not included in Round 2	Removed for stakeholders
**Consequences**			
3.15 Establish clearly stated exit mechanisms for partners	96 (96)	Not included in Round 2	77 (93)
3.16 Establish sanctions with effective enforcement for violation of conflict of interest including reprimands, fines, dismissal	91 (91)	Not included in Round 2	71 (85)
**4. Transparency**			
4.1 Explicitly report funding, governance structures, research frameworks and findings and ensure it is publically-available	98 (98)	Not included in Round 2	83 (99)
4.2 All individuals involved in a research partnership should undertake full disclosure including financial, personal and professional interests over the past 5 yrs	93 (93)	Not included in Round 2	80 (95)
4.3 All individuals involved in research partnership should disclose interests of their spouse/partner, minor children, employer and business partners	73 (73)	75 (82)	66 (79)
4.4 Ensure all presentations and media releases from an industry partner, regarding any research project to which they have contributed direct or in-kind funding, are endorsed by the research partner	77 (77)	79 (86)	66 (79)
4.5 Require full disclosure of funding sources and financial interests in research media releases	96 (96)	Not included in Round 2	83 (99)
4.6 Require a declaration of interests slide in all presentations and a written statement on any poster presentations	97 (97)	Not included in Round 2	82 (98)
4.7 Establish a public database of conflicts of interests in dietary public health research	86 (86)	Not included in Round 2	66 (79)
**5. Publication**			
5.1 Academic researchers should include all potential conflicts of interests, including full affiliation as well as disclosure of industry funding and/or industry affiliation in research publications)	59 (59)	90 (98)	83 (100)
5.2 Ensure research partner retains full rights to publish all results, including those unfavourable to the funder	98 (98)	Not included in Round 2	84 (100)
5.3 Ensure the research partner has control over the preparation and approval of peer-reviewed manuscript	98 (98)	Not included in Round 2	81 (96)
5.4 Establish clear definitions around sponsorships and author affiliations to be used in publications, such as: industry funded, non-industry-funded, and unknown/unclear sponsorship	99 (99)	Not included in Round 2	81 (96)
5.5 All conflicts of interest should be declared at the beginning of research articles in print and online	Not included in Round 1	82 (89)	77 (92)

**Key**: red = less than 60% agreement; orange = 60–79% agreement; green = more than 80% agreement

Of those taking part in round 1, 92 (92%) completed round 2. Consensus was achieved on an additional 11 statements. When combining the results from round 1 and round 2, 39 statements (68%) reached consensus (Table 2). A considerable amount of free text was provided by all participants in relation to individual principles and the principles overall.

#### Agreement—Stakeholder survey

The stakeholders had a similarly high level of agreement on many of the principles (Table 2). Of the 57 statements presented to stakeholders, there was 80% agreement or higher on 37 (65%) of these.

#### Statements with lowest levels of agreement

When examining the 23 statements that did not reach consensus either by researchers or stakeholders or both, just under half of these (n = 10) came close to the designated cut-off for consensus (i.e. ≥75% agreement). However, several remaining statements (n = 8) had substantially lower levels of agreement (i.e. <60%). Some of these statements had low levels of agreement between both researchers and stakeholders, whereas other statements elicited divergent responses across these two groups.

High levels of disagreement by both researchers and stakeholders. Several statements in the funding section had high levels of disagreement among both researchers and stakeholders included statement 1.2 *“a system where industry provides funding to research institutions*, *not individual researchers or research units*, *should be created”*. Qualitative feedback indicated that participants were generally very sceptical of this suggestion, stating that their institutions often have lower ethical standards than the participants themselves do, and that this process would not minimise conflicts of interest; it may in fact do the reverse. Strong disagreement was also seen for statement 2.3 which sought to restrict interaction to commercial partners who were committed to long-term engagement. Many participants stated that this was not a realistic principle and may result in valuable opportunities being missed. There was also a low level of agreement amongst both researchers and stakeholders with statement 1.5. This stated that researchers *“should not accept funds from commercial organisations”*. Most participants provided the caveat that their response related to funds from non-food and beverage companies.

The remaining two statements, which had similarly low levels of agreement between researchers and stakeholders, concerned attending industry-sponsored events and accepting in-kind funding. A number of different reasons for disagreeing with these statements were presented by participants (Table 3). Disagreement for some participants was due to the absolute nature of these statements, with several participants stating these decisions should be assessed on a case by case basis. Others believed there was value in presenting research findings at a food industry funded event as it may influence industry practices. Participants had stronger levels of agreement for declining in-kind funding from the food industry to present research findings at an event. However, for those who disagreed with this statement, some indicated that a blanket directive should not be made and it should depend -on the ‘healthfulness’ of the company’s products and actions. Others were concerned about the financial implications of such a statement, particularly in terms of being able to attend conferences.

**Table 3:** Comments from researchers and stakeholders on statements 2.15 and 2.16 concerning participation in events with food industry involvement.

**Statement 2.15 –Decline to give a presentation at events sponsored by the food industry**	
**Researchers (46% agreement)**	**Stakeholders (29% agreement)**
*A blanket ban on giving presentations is probably unhelpful*. *I think one should have the opportunity to give presentations that challenge the industry during industry events*. *On the other hand; giving presentations that align with industry interests may inflict reputational damage on the researcher; so one should think very carefully about accepting presentation invitations*. (**Researcher 64: Monitoring nutritional status & food environments, food/nutrition policy interventions, Malta**) *This is a really tricky one*. *I think it needs to be assessed on a case by case basis*. *I would definitely not accept funding to present; but if I self-funded to go and I thought it a really crucial event; I might consider it (although generally wouldn’t do it*). (**Researcher 12: Monitoring food environments, behavioural change interventions & reformulation, Australia**) *Some food companies are doing*, *or trying to do well by the public health*. *Even within the companies who are considered malevolent*, *there are often good people trying to do well by the public*. *The food industry should be approached as a potential partner in promoting the public’s health*: *e*.*g*. *getting McDonalds to make a healthier hamburger or other offerings can affect the 52 million meals they serve every day*: *what an impact on the public’s health* (**Researcher 138: Nutritional epidemiology, behavioural change interventions, USA**)	*Researchers shouldn’t give personally sponsored talks; but an event that takes sponsorship may occasionally be an important place to get the message across*. *Communicating only with other researchers and public health professionals is not always good enough*. (**Funder 12, UK**) *Difficult decision; many professional events for example professional events are sponsored by food industry; e*.*g*. *International Conference of Nutrition some years back was sponsored by Danone and Coca Cola*. *As long as you are independent; it maybe ok*. (**Policy officer 46, India**) *Without adequate funding from other sources*, *this is unrealistic*. *There is great value in dissemination of results across all sectors*. (**Policy influencer 80, USA**)
**Statement 2.16 –Decline funding (e.g. travel costs or honorarium) from the food industry to present research findings at an event**	
**Researchers (63% agreement)**	**Stakeholders (56% agreement)**
*Depends on the company and the healthfulness orientation of their products; as well as whether you are free to be critical even of items which would be similar to those they have in their portfolio*. (**Researcher: food/nutrition policy interventions + understanding food systems, Malta**) *Depends; but would be very careful*. *Very important to consider your reputation by doing that*. (**Researcher 23: Monitoring food environments + food/nutrition policy, Australia**) *This is simply impractical*: *academics have very limited budgets (I get £700 per year) and if we could not accept travel costs we simply could not attend*. *My expectation is that all costs are covered when giving an invited talk*. (**Researcher 93: Behavioural change interventions, food reformulation, UK**)	*Travel and accommodation; especially if not international and in standard class seem not unreasonable*. *Honoraria create an appearance of compromise*. (**Funder 14, UK**) *I’m really conflicted about this action*. *The reality is that covering travel costs is an enabler for PH researchers to get their information out there*. *But honorarium steps into a conflicted zone for me*. (**Policy influencer 22, Australia**) *Honorarium should be an industry standard amount and not an amount that would be used to influence the researcher*. (**Policy influencer 81, USA**)

Discordance between researchers and stakeholders. There were five statements where there were noticeable differences between researchers and stakeholders. These included stronger levels of agreement from stakeholders than researchers regarding the statement that population health researchers should not accept funds from the food industry, particularly the processed food industry. For these statements, emotive feedback was provided by participants within groups as well as between groups (Table 4). Some participants disagreed with the statements and highlighted that it was an important source of funding, while others found the blanket statements too broad to provide an opinion. Those who disagreed that it would be acceptable to receive funding from the food industry believed it provided little benefit for population health researchers and that it may lead to reputational damage. Others felt even more strongly about the ethical implications of interacting with the processed food industry likening it to engaging with tobacco companies.

**Table 4:** Comments from researchers and stakeholders on statements 1.3 and 1.4, concerning food industry funding for research.

**Statement 1.3 –Researchers should not accept funds from the food industry**	
**Researchers (43% agreement)**	**Stakeholders (70% agreement)**
*If appropriately managed and controlled to ensure no conflict of interest; it can be an important source of funding* (**Researcher 83: behavioural change interventions + develops methodologies for assessing/monitoring diet, South Africa**) *Depends on which is the company that is funding*. *if its products are against people’s health; I wouldn’t accept it (i*.*e*. *Coca cola)*. (**Researcher 51: nutritional epidemiology, Guatemala**)	*This is a broad statement and it depends on the type of food industry actor in question*. *If the industry actor is involved in producing unhealthy foods which are high in fat; salt and/or sugar then they should not fund research which could influence public health policy*. (**Policy influencer 13, UK**) *Considering the food industry as all actors of the food system; there may be some types of study that could receive funds; but with transparency*. (**Policy officer 30, Brazil**) *I think it depends on what company it is and what they are funding*. *I would object to companies that produce foods that are energy dense and nutrient poor*. *I would also object if there is a clear conflict of interest with what they are funding*. *However; not all companies produce these foods which is why I neither agree nor disagree*. (**Journalist 23, Australia**)
**Statement 1.4 –Researchers should not accept funds from processed food companies**	
**Researchers (55% agreement)**	**Stakeholders (80% agreement)**
*This becomes rather hard to define*, *as unhealthy and processed are not synonymous*. (**Researcher 81: monitoring food environments and diet, behavioural change interventions, South Africa**) *There is a whole range of processed food*. *One would need to be more specific as to what is not acceptable re ‘processed’*. (**Researcher 66: food/nutrition policy interventions + understanding food systems, Malta**) *The big food MNCs* [multi-national corporations] *produce commodities which kill; & deserve to be treated like tobacco MNCs*. (**Researcher 114: food/nutrition policy, nutritional epidemiology, UK**)	*This might be ideal; but not practical at present; so a ban is not reasonable*. *Disclosure is critical*. (**Policy influencer 56, USA**) *It depends on the kind of research*. *If it is on a product that the food industry has an interest in; they should not*. (**Policy Officer 52, Italy**) *I guess "processed" is meant to mean unhealthy—although that isn’t always the case*. *A lot depends on the context; but it’s rarely a good idea*. (**Funder 12, UK**)

The categorisation of companies in terms of acceptability of receiving funding from, or interacting in other ways with the food industry, was a frequent source of concern and confusion for participants. This was evident from the responses to the three funding statements (1.3–1.5) as well as statement 2.8 ‘*Avoid companies whose objectives and/or goals are related to the increased production*, *supply or demand of ‘unhealthy food’ products and/or to the promotion of unhealthy and unsustainable ways of eating and producing food’*. While high levels of agreement were seen for statement 2.8, it was challenging for many participants to define the types of companies they considered acceptable for researchers to interact with (Table 5). Not all participants believed these companies should be avoided and that there was even a responsibility and/or an opportunity for them to be involved in ‘fixing’ the problem.

**Table 5:** Comments from researchers and stakeholders on statement 2.8, concerning unhealthy food companies.

Statement 2.8 –Avoid companies whose objectives and/or goals are related to the increased production, supply or demand of ‘unhealthy food’ products and/or to the promotion of unhealthy and unsustainable ways of eating and producing food	
Researchers (75% agreement)	Stakeholders (82% agreement)
*Almost all food companies are good and bad*. *This would rule out work with all supermarkets; for example*. (**Researcher 77: Nutritional epidemiology, behavioural change interventions + food/nutrition policy, Pakistan**) *There are instances when they are the companies you would want to be working with*. *They are the companies who have created the whole problem in the first place; so they’re the ones that are most in need of change*… (**Researcher 15: monitoring food environments + behavioural change interventions, Australia**) *Preferably not; because your credibility is in doubt once you’ve personally accepted industry funding*. (**Researcher 152: Monitoring food environments, behavioural change interventions, food/nutrition policy, New Zealand**)	*The definition of healthy versus unhealthy can and will constantly be debated; and companies may be on an improvement trajectory that should not preclude their involvement; and many/most large companies produce both healthy and unhealthy products*. (**Policy influencer 80, USA**) *This is the crux of the issue*. *Most companies in a position to be funders of research will likely have a product portfolio that still contains a significant amount of HFSS/non-core foods that account for significant sales value (even if not the absolute majority of products in the portfolio)*. *This applies as much to "conservative" companies as to those that could argue they are "first movers" towards healthier foods*. *If the research funding role somehow gives them a halo and access among research funders and policy-makers; this could be problematic for driving research attention and policy change in areas that target the HFSS/non-core foods*. (**Policy officer 35, Denmark**) *Agree in general; but some companies; such as retailers; can have objectives which align to BOTH healthy and unhealthy diets*. (**Funder 12, UK**)

The remaining two statements with low levels of accordance between researchers and stakeholders were 2.2 and 3.5. Statement 3.5 focused on using independent statisticians for data analysis. Feedback from participants on this item highlighted pragmatic considerations, with lack of funding to employ additional staff cited as a key reason for not agreeing with the statement. Compared to researchers, stakeholders were much more likely to disagree with statement 2.2 *“a partnership should only be initiated if it will help advance the public health goal”*. Comments indicated this disagreement was driven by scepticism that the food industry would want to advance public health goals and a concern for conflicts of interest that may arise. There was also concern about how different groups might interpret the phrase *“advance the public health goal”*.

## Discussion

### Main findings in the context of existing knowledge

This international study aimed to determine levels of agreement, amongst researchers and research stakeholders, with principles for preventing or managing conflicts of interest between population health researchers and the food industry. While previous studies have looked at the attitudes of researchers towards industry involvement in research,[26, 27] this is the first study, to our knowledge, to seek consensus among population health researchers and level of agreement among research stakeholders internationally on the principles for preventing or managing conflicts of interest between population health researchers and the food industry.

High levels of agreement were reached for many of the statements in the Delphi study, with consensus (at least 80% agreement among participants) achieved on 68% of all statements presented in round 2. Similarly, research stakeholders showed high levels of agreement on 69% of the principles. In common with the scoping review that informed this Delphi study, the principles that attracted the highest levels of agreement were those that were derived from widely accepted research governance frameworks and involve limited values-based decision making. This is in accordance with previous Delphi research that has shown the natural tendency of Delphi studies to rank highly those items that are non-controversial.[28]

The most contentious statements, both in terms of agreement levels and emotive comments from participants, were concerned with which elements of the commercial sector it is acceptable for population health researchers to interact. Most researchers and stakeholders believed it was appropriate for population health researchers to accept funding from commercial organisations in general. However, the level of agreement decreased when statements specified accepting funding from the food industry and, in particular, processed food companies. This is unsurprising, as many see the primary goal of population health researchers (to discover new knowledge to improve the health of the population) as being poorly aligned with the primary goal of most food companies (to generate profit).[10, 11, 27, 29] Furthermore, recommendations from population health researchers frequently encourage government actions that are contrary to the preferences of the commercial sector. Interestingly, stakeholders expressed stronger views than researchers on the subject of not engaging with the food industry. This may be a consequence of the range of researchers involved in the Delphi study, which included those who currently interact with the food industry who may have an interest in continuing food industry interactions.

In common with previous research that has focused on academics generally, we found there was strong support for full disclosure of interests and the application of rigorous research governance principles.[30, 31] This supports the view that, if individuals are open and do not engage in obvious misconduct, no harm will result.[31] However, while full disclosure of interests is acknowledged as an important step, previous research has demonstrated that disclosure on its own is not enough to effectively prevent or manage conflicts of interest and, in fact, may worsen the situation in some instances.[32, 33] This sentiment was noted by many participants, with concerns about perceived conflicts of interest and the reputational damage that could result from interactions with the food industry. Rather than concerns about unconscious bias or the corporate capture of research topics, which have been identified as problems in the literature,[8, 10] many participants indicated that risk of reputational damage was the key challenge of interacting with the food industry.

### Strengths and weaknesses of the study

Our sample size for the Delphi survey was higher than the average number typically included in a Delphi study targeting population health researchers.[34] However it was not large enough to conduct sub-group analysis, for example, by country. Despite this limitation, our sample included researchers and stakeholders from a wide range of backgrounds with high levels of experience. This gives us confidence that we have captured the range of opinion available on this issue. Our 92% response rate for round two was a strength, as Delphi studies are typically more demanding for participants than a simple survey. This high response rate decreases the risk of response bias.[22] A further strength of our study was the considerable levels of experience reported by the majority of participants.[21]

Using an online method to collect data allowed anonymous participation and therefore encouraged free expression of opinions. It also allowed the inclusion of a diverse range of researchers from around the world to participate. In addition, as this topic can provoke emotive responses, the online method enabled consistent and fair consideration of all participants’ opinions, without the risk of domination by strong characters, such as might occurs in a face to face encounter.[35] Using participant feedback from round 1 to refine and clarify the statements resulted in greater understanding and higher levels of agreement in round 2. We also saw the benefits of a two-stage Delphi survey in encouraging participants to move from a neutral standpoint in the first round to taking a position on a statement in round two, aided by seeing the level of agreement of all participants from round one.

One of the limitations of this study is the arbitrarily set cut-off point for consensus. However, a higher level of agreement (80%) than in other Delphi studies was chosen as we felt it was important for this controversial issue. It is important to recognise that consensus is not the same as unanimity, and there were statements that reached high levels of consensus but still provoked strong feelings among some participants. Another limitation was the selection process for participants. To minimise the potential for selection bias, we established clear criteria for including experts at the outset. However, due to the recruitment process we are unable to report on response rate of participants and cannot claim that the researcher or stakeholder samples were representative of population health researchers or related stakeholders in general. Further work could explore whether and how other stakeholders not included here should be engaged in developing and applying consensus. A further limitation was that the survey was in English and so we may have missed the opinions of participants from other countries where English is not their primary language. This may have had differential effects in high, middle and low-income countries.

### Meaning of the study: Possible explanations and implications for practitioners and policymakers

Our findings suggest that researchers and associated stakeholders are generally highly supportive of principles that were focussed on research methods and governance that are already captured in existing research governance frameworks. Principles for which there were lower levels of agreement were often accompanied by emotive comments from participants, or confusion over what was the ‘right’ decision. Competing priorities appeared to underpin much of this confusion and emotion. For example, we observed tensions between scientific expertise and peer or public opinion; and between job security and integrity. These competing priorities and associated internal struggles faced by population health researchers illuminate the moral or ethical nature of these issues. Practical guidance that acknowledges the moral challenges of the issues and has the support of a wide range of stakeholders, including journals and research funders needs to be developed to help researchers navigate this complex issue.

### Unanswered questions and future research

The study provides vital information that will underpin the development of guidance and associated tools to help researchers navigate this challenging set of issues. However, in doing so, it has also raised a number of questions requiring further research. The participants expressed a desire for greater clarity regarding definitions of the terms used in the Delphi study statements. In particular, these concerned the categorisation of a heterogeneous food industry. The findings revealed differing views on the complex issues of preventing and managing conflicts of interest, and it was clear that experience of these issues varies greatly across different nations and cultures. Further research to understand better the knowledge and experience of these issues, and the ways they are handled in low- and middle-income countries in particular, may help to assess the barriers to implementing guidelines in the future. There is also a need to discern the similarities and differences between our findings and those other research fields, such as biomedical and pharmaceutical research, where industry research collaborations are widespread, and the goals of industry and research are often more closely aligned. Such collaborations are actively promoted in many countries, for example in the UK by the Government’s Industrial and Life Sciences Strategies.[36] Finally, further quantitative analysis could be conducted on this data to further understand the differences of opinion between researcher and stakeholders and the different groups within these categories.

## Conclusions and implications

Overall, a high level of agreement among population health researchers and research stakeholders was achieved for most of the principles presented in the two surveys on preventing and managing conflicts of interest in interactions with the food industry. The principles that had the lowest level of agreement related to which companies population health researchers consider it is appropriate to interact with, whether through accepting direct funding, attending industry sponsored events or accepting in-kind funding. It is important to note that the Delphi process, despite building consensus, does not necessarily lead to the ‘correct’ answer. It merely leads to a certain level of agreement with the statements that have been provided. With this in mind, further exploration of this issue, in particular categorisation of the food industry and the development of a risk assessment tool will provide greater insight and clarity into this issue.

The results of this study provide the basis for developing internationally agreed guidance for population health researchers, governing interactions with the food industry. Such guidance will need to be supported by risk assessment tools for researchers, which could build on existing materials developed for non-government organisations [37] and countries considering food sector partners in nutrition policy initiatives.[6] However, such risk assessment tools need to be tested for appropriateness with researchers. The research has also generated consensus on many principles that may be transferrable to other research fields and may help thinking more broadly about the issue of managing conflicts of interest between scientists and commercial or other vested interests.

## Supporting information

S1 File Authors declaration of interests. (DOCX)

S2 File Delphi_round 2 sample survey form. (PDF)

S3 File Survey round 1 sample form. (PDF)
