# Supplementary material for: Building consensus on interactions between population health researchers and the food industry: Two-stage, online, international Delphi study and stakeholder survey
Source: PLoS One. 2019 Aug 22;14(8):e0221250. doi: 10.1371/journal.pone.0221250 (PMC6705832; doi:10.1371/journal.pone.0221250)
Supplement: S1 File — (DOCX) [file pone.0221250.s001.docx]

**S1: Author’s declaration of interests**

**Dr Katherine Cullerton**

***Research support***

The MRC Epidemiology Unit has received a grant from the UK Medical Research Council (PI Martin White) to undertake this research.

***Non-financial support***

The MRC Epidemiology Unit provides the premises and facilities that has supported this research to be undertaken. In previous research, I have conducted in-depth interviews with food industry senior executives regarding their influence on nutrition policy. All participants gave their time freely.

***Public statements and positions***

I have provided expert testimony to a House of Representatives Committee inquiry into remote Aboriginal and Torres Strait Islander community food stores.

***Non-financial interests***

I am a member of the executive committee for the Food and Nutrition Special Interest Group (FANSIG) of the Public Health Association of Australia. FANSIG has advocated for public health interventions to promote healthier diet, including regulation of commercial food companies.

***Additional information***

My partner works in the advertising industry, previously as a creative director, now as a consultant. He has not worked for a food or beverage client in over three years and I have no involvement with his work.

**Dr Jean Adams**

***Appointments & activity***

I sit on Public Health England’s Nutrient Profile Model expert review group. The NPM is used to restrict food advertising to children in the UK.

***Employment & Consulting***

I am a member of Cancer Research UK’s prevention expert review group. I receive an honorarium for every meeting I attend. CRUK advocate for a range of nutrition policies.

***Research support***

I hold research grants from NIHR, the Health Foundation, Public Health England, and the Economic and Social Research Council. My salary is supported by an infrastructure grant administered by the Medical Research Council.

***Non-financial support***

None beyond paid travel for activities reported above.

**Investment interests**

I hold an ‘ethical’ investment portfolio with the Co-operative Bank. The companies included are reviewed and selected by the bank, not me.

***Public statements and positions***

My research unit has provided evidence to the UK House of Commons Health Select Committee on diet and physical activity research. I have provided direct expert testimony to the same committee and to the British-Irish Parliamentary Assembly. I have submitted written responses to various parliamentary and other consultations on food and nutrition policy in a professional capacity. I have contributed to evidence submissions related to appeals when planning permission for new hot food takeaways have been denied. See my statement about Public Health England’s NPM review group above.

***Non-financial interests***

I am an academic editor of International Journal of Behavioural Nutrition and Physical Activity.

***Any other information***

My partner, Martin White, is a co-author.

**Mr Oliver Francis**

***Research support***

The MRC Epidemiology Unit has received a grant from the UK Medical Research Council (PI Martin White) to undertake this research. My salary is funded via a grant to the Centre for Diet and Activity Research (CEDAR) in the MRC Epidemiology Unit, University of Cambridge. CEDAR is funded by BHF, CRUK, ESRC, MRC, NIHR and The Wellcome Trust.

***Non-financial support***

The MRC Epidemiology Unit provides the premises and facilities that has supported this research to be undertaken.

**Prof Nita Forouhi**

***Research Support***

I have received grant funding from the Medical Research Council and the European Union FP6 and FP7 programmes. My salary is funded via a MRC grant to the MRC Epidemiology Unit, and I also receive part funding from the NIHR Biomedical Research Centre Cambridge: Nutrition, Diet, and Lifestyle Research Theme.

***Public statements and positions***

I contributed as committee member for the BMJ on a series of 12 articles on the science and politics of food and health. This series was sponsored for open access publication by Swiss Re, a re-insurance company. The Swiss Re Institute hosted a conference to launch the series and paid my travel and accommodation costs in June 2018. I have advised Diabetes UK on some of its position statements, and was peer reviewer for the DUK dietary guidelines released in 2018. I contribute regularly to statements in the journalistic media on diet and health related topics, through the print, online and broadcast media.

***Appointments & activity***

I am a member of Expert Group of an ILSI-Europe task force on qualitative fat intake: Update on Health Effects of Different Dietary Saturated Fats. ILSI-Europe is a not-for-profit agency that includes partnership between public, private and academic institutions. I have not received any funds or honoraria or travel fees. I have provided academic input to the work.

***Non-financial interests***

The MRC Epidemiology Unit provides the premises and facilities that has supported this research to be undertaken. I am a Fellow of the UK Faculty of Public Health (FPH) of the Royal College of Physicians of London. FPH has advocated for public health interventions to promote healthier diet, including regulation of commercial food companies. My research on dietary factors and health has been cited by evidence reviews undertaken by the UK Scientific Advisory Committee on Nutrition (SACN) and the World Health Organization (WHO), and has been part of the narrative in strategies that led to the implementation of a sugary drinks industry levy.

I am an expert member of the joint SACN/NHS_England/Diabetes_UK Working Group on lower carbohydrate diets and type 2 diabetes.

I have served roles as an expert witness and co-opted invited member to NICE and NICE Public Health Interventions Advisory Committee (PHIAC). I am also an adviser for the NICE Centre for Guidelines (CfG).

I am a member of Expert Group of an ILSI-Europe task force on qualitative fat intake: Update on Health Effects of Different Dietary Saturated Fats. ILSI-Europe is a not-for-profit agency that includes partnership between public, private and academic institutions. I have not received any funds or honoraria or travel fees. I have provided academic input to the work via telephone meetings.

**Prof Martin White**

***Research Support***

The MRC Epidemiology Unit has received a grant from the UK Medical Research Council (PI Martin White) to undertake this research.

I receive grant funding from a range of funders including NIHR, DH and MRC related to understanding the impact of interventions on diet and diet-related ill-health. My salary is funded via a grant to the Centre for Diet and Activity Research (CEDAR) in the MRC Epidemiology Unit, University of Cambridge. CEDAR is funded by BHF, CRUK, ESRC, MRC, NIHR and The Wellcome Trust.

***Non-financial support***

The MRC Epidemiology Unit provides the premises and facilities that has supported this research to be undertaken.

***Non-financial interests***

I am a member of the UK Faculty of Public Health of the Royal College of Physicians of London. FPH has advocated for public health interventions to promote healthier diet, including regulation of commercial food companies.

I am Director of the National Institute for Health Research’s Public Health Research Programme, which funds research to underpin non-health sector public health interventions (including dietary public health interventions). This position pays my salary for one day/week.

I am a member of the Medical Research Council’s Population Health Sciences Group, which provides strategic advice to the MRC Strategy Board. In common with other government research funders, MRC encourages co-funding of research with commercial companies. It has recently developed a new nutrition research strategy, which advocates collaboration with the food industry. It has also recently developed draft guidance for researchers on managing competing interests in relationships with commercial partners. MRC requires all staff and members of committees to declare their conflicts of interest, which are publicly available.

I am an expert advisor to the Food Foundation, a non-governmental charitable organization that advocates for healthier diets and food systems for all. I am Leader within the Centre for Diet and Activity Research for a programme of research on food systems and public health. This focuses primarily on understanding commercial food systems with a view to achieving closer alignment with public health goals.

***Any other information***

My partner, Jean Adams, is a co-author.
