# Supplementary material for: Building consensus on interactions between population health researchers and the food industry: Two-stage, online, international Delphi study and stakeholder survey
Source: PLoS One. 2019 Aug 22;14(8):e0221250. doi: 10.1371/journal.pone.0221250 (PMC6705832; doi:10.1371/journal.pone.0221250)
Supplement: S2 File — (PDF) [file pone.0221250.s002.pdf]

After agreeing to participate, I was provided with a website address and unique ID and password to log into the online survey for this study. By logging into the system, and ticking these boxes I confirm that:

☐ I have read and understood the Participant Information Sheet version 1 dated 23/5/2017 and have had the opportunity to ask questions and have had them answered.

☐ I understand that all personal information will remain confidential and that all efforts will be made to ensure that I cannot be identified (except as might be required by law).

☐ I agree that data gathered in this study will be stored anonymously and securely, and may be used in future research.

☐ I understand that my participation is voluntary and that I am free to withdraw at any time without giving a reason.

If you have any questions about these consent details, please contact coi@mrc-epid.cam.ac.uk.

Preventing and Managing Food Industry Conflicts of Interest

Research Consensus

Responding to the lack of explicit consensus about what constitutes acceptable or effective engagement between researchers and the food industry, the Centre for Diet and Activity Research (CEDAR) at the University of Cambridge has funded a project to explore and seek consensus on this issue by undertaking a Delphi survey.

For round 2 of the Delphi survey we would like you to rate the statements on the following pages based on your personal opinion. In most cases you will see your previous response but can change this by clicking the relevant button. This round of the survey will only examine the general principles and actions for preventing and managing conflicts of interest between dietary public health researchers and the food industry that did not reach high levels of agreement in the first round. Please check the statements and consider carefully whether you would like to change your response.

In this survey commercial food/and or beverage companies will be referred to generically as the "food industry" for brevity. This term encompasses growers, producers, manufacturers, distributors, retailers and their representative associations that range in size from small enterprises to large multi-national corporations. We exclude from this definition the alcohol industry, although note that some companies are involved in both food and alcohol.

In this survey, we also refer to "researchers", by which we mean those whose research includes a focus on diet or nutrition and health. Our particular interest is in dietary public health and public health nutrition research.

Question 1

This question is exploring different parts of the research process. There are five sections ranging from funding to publication. In each section, please rate **how strongly you agree with the stated actions to *prevent* or *manage* conflicts of interest** between dietary public health researchers and the food industry.

Please rate each action as it is stated based on your personal opinion. You have the opportunity to suggest modifications beside each statement or add new actions in a text box at the end of the question. The statements have been generated by a systematic review of the published and grey literature on preventing and managing conflicts of interest in this area. Please note changes to the statements from round 1 are identified in red and, in some cases, this has resulted in completely new statements.

**Statement**	**Rating**					**Comment**
	**Strongly Agree**	**Agree**	**Neither agree or disagree**	**Disagree**	**Strongly disagree**	
**Funding**						
Q1) A pool of funding from the food industry which is independently administered by a publically accountable third party should be created [74% agreement]						
Q2) A system where industry provides funding to research institutions, not individual researchers or research units, should be created [34% agreement]						
Q3) Researchers should not accept funds from the food industry [47% agreement]						
Q4) Researchers should not accept funds from processed food companies [53% agreement]						
Q5) Researchers should not accept funds from any commercial organisation [24% agreement]						
**For those who accept funding from the food industry**						
Q2) Funding from industry should reflect the full cost of the research (eg using standard academic costing) and not more than this amount [70% agreement]						
Q3) Industry funding should be non-designated [70% agreement]						
Q4) There should be no involvement of a food industry funder in any aspect of a research project [70% agreement]						

Q1b Undertake thorough Risk Assessment	Rating					Comment
	Strongly Agree	Agree	Neither agree or disagree	Disagree	Strongly disagree	
Q2) A partnership should only be initiated if it will help advance the public health goal [74% agreement]						
Q3) Only enlist partners who are committed to long term funding and engagement [35% agreement]						
Q4) Only enlist partners who are committed to sharing of research data arising from the research project [77% agreement]						
Q7) Ensure all partners have shared objectives and a shared approach to the research question and activities [77% agreement]						
Q8) Avoid companies whose objectives and/or goals are related to the increased production, supply or demand of 'unhealthy food' products and/or to the promotion of unhealthy and unsustainable ways of eating and producing food [76% agreement]						
Risk Assessment of type of engagement						
Q1) Consider whether the proposed engagement would be acceptable across institutions and national borders [68% agreement]						
Consider possibility of reputational damage and loss of trust						
Q1) Consider what my colleagues would think about this arrangement [71% agreement]						
Q2a) Decline to give a presentation at events sponsored by the food industry						
Q2b) Decline funding (e.g. travel costs or honorarium) from the food industry to present research findings at an event						
Q6) Do not allow the commercial partner to co-brand (e.g. use their logo) on the research project or related material [77% agreement]						
Research governance						
1a) Establish up-front control and ownership of the data by the researcher/s irrespective of the funding source						
1b) Provide open access to anonymised data and analyses once results are published						
2) Data analysis should be done by statisticians independent of the researcher/s who designed and conducted the study [52% agreement]						

3) Undertake random audits of data provided by food companies for research projects [76% agreement]						
4) Secure oversight of the research by a non-conflicted third party [74% agreement]						
**Ensure partners have equal power**	**Strongly Agree**	**Agree**	**Neither agree or disagree**	**Disagree**	**Strongly disagree**	**Comment**
1) If the food industry is supporting research by providing direct funding or data, ensure they do not have undue influence over research by having a diversity of partners on project steering committees (e.g. foundations, NGOs, consumers).						
**Ensure public benefit is at centre of agreement**						
1) Engage independent members of the public in the process of defining research problems and subjecting research projects to ongoing critical scrutiny [71% agreement]						
Transparency						
1) All individuals involved in research partnership should disclose interests of their spouse/partner, minor children, employer and business partners [73% agreement]						
2) Ensure all presentations and media releases from an industry partner, regarding any research project to which they have contributed direct or in-kind funding, are endorsed by the research partner [77 % agreement]						
**Publication**						
1) Academic researchers should include all potential conflicts of interests, including full affiliation as well as disclosure of industry funding and/or industry affiliation in research publications [59% agreement]						
2) All conflicts of interest should be declared at the beginning of research articles in print and online						

Thank you for participating in the second round of this survey. Upon completion of this study, we will send you a summary of the results by email. We will also notify you of any publications arising from the research and future work.

If you have any questions, please contact Katherine Cullerton on coi@mrc-epid.cam.ac.uk
