# Supplementary material for: Building consensus on interactions between population health researchers and the food industry: Two-stage, online, international Delphi study and stakeholder survey
Source: PLoS One. 2019 Aug 22;14(8):e0221250. doi: 10.1371/journal.pone.0221250 (PMC6705832; doi:10.1371/journal.pone.0221250)
Supplement: S3 File — (PDF) [file pone.0221250.s003.pdf]

Consent

COI (v. 1_11/04/2017)

Principal Investigator

Professor Martin White
Programme Leader for Research in Food Behaviours & Public Health
MRC Epidemiology Unit
University of Cambridge

After agreeing to participate, I was provided with a website address and unique ID and password to log into the online survey for this study. By logging into the system, and ticking these boxes I confirm that:

- [x] I have read and understood the Participant Information Sheet version 1 dated 23/5/2017 and have had the opportunity to ask questions and have had them answered.
- [x] I understand that all personal information will remain confidential and that all efforts will be made to ensure that I cannot be identified (except as might be required by law).
- [x] I agree that data gathered in this study will be stored anonymously and securely, and may be used in future research.
- [x] I understand that my participation is voluntary and that I am free to withdraw at any time without giving a reason.

If you have any questions about these consent details, please contact coi@mrc-epid.cam.ac.uk.

Please note, you are able to complete part of the survey and finish it at a different time. However, please complete the whole page and click "Next" for your answers up to that point to be saved.

COI (v. 1_11/04/2017)

In this survey commercial food/and or beverage companies will be referred to generically as the "food industry" for brevity. This term encompasses growers, producers, manufacturers, distributors, retailers and their representative associations that range in size from small enterprises to large multi-national corporations. We exclude from this definition the alcohol industry, although note that some companies are involved in both food and alcohol.

In this survey, we also refer to "researchers", by which we mean those whose research includes a focus on diet or nutrition and health. Our particular interest is in dietary public health and public health nutrition research.

Do you currently work (paid employment) for the food industry?

- ○ Yes
- ● No

Do you currently undertake, review or publish research as part of your employment?

- ● Yes
- ○ No

Does this research include a focus on food, diet and/or nutrition and health?

- ● Yes
- ○ No

COI (v. 1_11/04/2017)

What type of research do you primarily undertake?
Feel free to tick more than one

- [ ] Monitoring and surveillance of nutritional status
- [ ] Monitoring and surveillance of food environments
- [ ] Nutritional epidemiology
- [ ] Development or evaluation of behavioural change interventions to improve change diet/nutritional status of populations
- [x] Development, analysis or evaluation of food or nutrition policy interventions
- [ ] Food composition/reformulation
- [ ] Understanding and/or changing appetite
- [ ] Understanding and/or changing food systems
- [ ] Developing or evaluating methodologies needed for assessment or monitoring of diet
- [ ] Development or evaluation of interventions to communicate nutrition-related information
- [ ] Laboratory-based research on nutrients or related biomarkers
- [ ] Other food, diet or nutrition related research (please explain below)

What type of organisation is your main employer?

- (●) Higher education institution (e.g. university or college)
- ( ) Government or public sector organisation
- ( ) Non-governmental organisation (e.g. voluntary or charitable organisation)
- ( ) Academic publishing organisation
- ( ) Other organisation (please explain below)

Please state the country you are currently employed in? UK

How long have you been working in the field of food, diet and/or nutrition and health?

- ( ) 1-5 yrs
- ( ) 5-10 yrs
- (●) 10+ yrs

COI (v. 1_11/04/2017)

In the *past 5 years*, have you accepted direct funding (e.g. research grants, fellowships) from the food and/or beverage (not alcohol) industry?

- ○ Yes
- ● No

Have you ***ever*** accepted direct funding (e.g. research grants) from the food industry?

- ○ Yes
- ● No

In the *past 5 years*, have you accepted in-kind contributions (e.g. conference fees, travel costs) from the food industry?

- ○ Yes
- ● No

Have you ***ever*** accepted in-kind contributions (e.g. conference fees, travel costs) from the food industry?

- ○ Yes
- ● No

In the *past 5 years*, have you engaged in formal dialogue (e.g. exchanging information or data and/or providing guidance or advice without receiving financial contributions) with the food industry?

- ○ Yes
- ● No

Have you ***ever*** engaged in formal dialogue (e.g. exchanging information and/or providing guidance or advice, without receiving financial contributions) with the food industry?

- ○ Yes
- ● No

Have you had any other interaction with the food industry or affiliated organisations including employment, that is not described above?

- ● Yes
- ○ No

Please describe the interactions

xogfgf

Introduction *COI (v. 1_11/04/2017)*

Preventing and Managing Food Industry Conflicts of Interest
Research Consensus

Responding to the lack of explicit consensus about what constitutes acceptable or effective engagement between researchers and the food industry, the Centre for Diet and Activity Research (CEDAR) at the University of Cambridge has funded a project to explore and seek consensus on this issue.

As part of this project we would like you to rate the statements associated with the three questions on the following pages based on your personal opinion. The first question examines general principles and actions for preventing and managing conflicts of interest between researchers and the food industry; these actions have previously been identified by researchers and different health associations. Questions 2 and 3 explore further the types of food industry organisation(s) that it may be acceptable to engage with.

Question 1

COI (v. 1_11/04/2017)

This question is exploring different parts of the research process. There are five sections ranging from funding to publication. In each section, please rate **how strongly do you agree with the stated actions to *prevent* or *manage* conflicts of interest** between researchers and the food industry.

Although some of the actions may seem similar, please rate each action as it is stated based on your personal opinion. You have the opportunity to suggest modifications beside each statement or add new actions in a text box at the end of the question. The statements have been generated by a systematic review of the published and grey literature on preventing and managing conflicts of interest in this area. You may need to scroll down the page to see all the statements.

Actions	Rating					Comment
	Strongly agree	Agree	Neither agree or disagree	Disagree	Strongly disagree	
**Funding**						
A pool of funding from the food industry which is independently administered by a third party should be created	●	○	○	○	○	
A system where industry provides funding to research institutions, not individual researchers or research units, should be created	●	○	○	○	○	
Researchers should not accept funds from the food industry	●	○	○	○	○	
Researchers should not accept funds from processed food companies	●	○	○	○	○	
Researchers should not accept funds from any commercial organisation	●	○	○	○	○	
**For researchers who do accept funding from the food industry**						
Researchers should have no commercial interest in the product being researched	●	○	○	○	○	
Funding from industry should reflect the	●	○	○	○	○	

cost of the research and not more than this amount						
Industry funding should be non-designated (i.e. use of funding is not determined by the funder)	●	○	○	○	○	
There should be no involvement of the funder in any aspect of project	●	○	○	○	○	
There should be limited involvement of the funder in any aspect of the project	●	○	○	○	○	

Question 1 (continued)

COI (v. 1_11/04/2017)

Actions	Rating					Comment
	Strongly agree	Agree	Neither agree or disagree	Disagree	Strongly disagree	
**Undertake thorough Risk Assessment**						
**Risk Assessment of potential commercial partner (which may or may not include funds)**						
Have a clearly identified system to identify and assess interests of potential partners	●					
A partnership should only be initiated if it will help advance the public health goal	●					
Only enlist partners who are committed to long term funding and engagement	●					
Only enlist partners who are committed to sharing of research data	●					
Only enlist partners who operate in an ethical manner and uphold the human rights of women, men and children	●					
Ensure the organisational values and overarching goals of the partners are compatible	●					
Ensure all partners have shared objectives and a shared approach	●					
Avoid companies whose objectives and/or goals are related to the increased production, supply or demand of 'unhealthy food' products and/or to the promotion of unhealthy and unsustainable ways of eating and producing food	●					

Assess whether the partnership could undermine the integrity or trustworthiness of my institution	●	○	○	○	○	
Risk Assessment of type of engagement						
Conduct comparative assessments across institutions and national borders to determine whether the proposed engagement would be acceptable	●	○	○	○	○	
Be guided by generic international protocols and frameworks (e.g. World Health Organisation) on appropriate types of engagement	●	○	○	○	○	
Ensure public benefit is at centre of agreement						
Consider whether the partnership provides maximum benefit to society	●	○	○	○	○	
Consider what the public would think about this arrangement	●	○	○	○	○	
Consider possibility of reputational damage and loss of trust						
Consider what my colleagues would think about this arrangement	●	○	○	○	○	
Decline to give industry sponsored presentations	●	○	○	○	○	
Do not 'ghost-write' publications for the private sector	●	○	○	○	○	
Do not accept gifts or hospitality if it compromises or appears to compromise objectivity	●	○	○	○	○	
Do not participate in undisclosed paid authorship arrangements in industry-sponsored publications or presentations	●	○	○	○	○	

Do not allow the commercial partner to brand any project or related material	●	○	○	○	○	

Question 1 (continued)

COI (v. 1_11/04/2017)

Actions	*Rating*					*Comment*
	Strongly agree	*Agree*	*Neither agree or disagree*	*Disagree*	*Strongly disagree*	
Research governance						
Clearly state and agree on goals, objectives, roles and responsibilities and accountability before work commences	●	○	○	○	○	
Plan research so it is designed objectively and is scientifically sound in its approach	●	○	○	○	○	
Establish up-front control and ownership of the data by the researcher/s but provide accessibility to data and analyses to the industry funder	●	○	○	○	○	
Data analysis should be done by statisticians independent of the researcher/s who designed and conducted the study	●	○	○	○	○	
Undertake random audits of data provided by food companies	●	○	○	○	○	
Secure oversight of the research by a non-conflicted third party	●	○	○	○	○	
Require all trials or other evaluative studies in dietary public health to be registered at time of initiation of the study	●	○	○	○	○	
Ensure partners have equal power						
Along with the commercial sector, include members of civil society (e.g. foundations, NGOs, consumers) as partners	●	○	○	○	○	

Ensure diversity of partners to avoid undue influence of any one partner	●	○	○	○	○	
The research institution must be able to independently criticize a commercial-sector entity for issues unrelated to the partnership	●	○	○	○	○	
Ensure public benefit is at centre of agreement						
Engage members of the public in the process of defining research problems and subjecting research projects to ongoing critical scrutiny	●	○	○	○	○	
Management of conflict(s) of interest						
Have a clearly identified system to identify, assess and manage the interests of all stakeholders	●	○	○	○	○	
Recuse stakeholders from committee (or similar) decision making where there may be an actual or perceived conflict	●	○	○	○	○	
Continuously monitor for conflicts of interest	●	○	○	○	○	
Consequences						
Establish clearly stated exit mechanisms for partners	●	○	○	○	○	
Establish sanctions with effective enforcement for violation of conflict of interest including reprimands, fines, dismissal	●	○	○	○	○	

Question 1 (continued)

COI (v. 1_11/04/2017)

Actions	***Rating***					***Comment***
	Strongly agree	*Agree*	*Neither agree or disagree*	*Disagree*	*Strongly disagree*	
Transparency						
Explicitly report funding, governance structures, research frameworks and findings and ensure it is publically-available	●	○	○	○	○	
All individuals involved in a research partnership should undertake full disclosure including financial, personal and professional interests over the past 5 yrs	●	○	○	○	○	
All individuals involved in research partnership should disclose interests of their spouse/partner, minor children, employer and business partners	●	○	○	○	○	
Ensure all presentations and media releases from an industry partner are endorsed by the research partner	●	○	○	○	○	
Require full disclosure of funding sources and financial interests in research media releases	●	○	○	○	○	
Require a declaration of interests slide in all presentations and a written statement on any poster presentations	●	○	○	○	○	
Establish a public database of conflicts of interests in dietary public health research	●	○	○	○	○	

Question 1 (continued)

COI (v. 1_11/04/2017)

Actions	*Rating*					*Comment*
	Strongly agree	*Agree*	*Neither agree or disagree*	*Disagree*	*Strongly disagree*	
Publication						
Ensure academic researchers, when they act as contract researchers, publish only under the name of the contract research organisation/s	●	○	○	○	○	
Ensure the research partner retains full rights to publish all results, including those unfavourable to the funder	●	○	○	○	○	
Ensure the research partner has control over the preparation and approval of peer-reviewed manuscript	●	○	○	○	○	
Establish clear definitions around sponsorships and author affiliations to be used in publications, such as: industry funded, non-industry-funded, and unknown/unclear sponsorship	●	○	○	○	○	

Please feel free to add below any principles you feel are important that have not been identified above

Question 2

COI (v. 1_11/04/2017)

The previous question examined some characteristics of a commercial food and/or beverage company that you may consider when assessing whether or not you should engage with them. Are there any additional characteristics of a commercial food and/or beverage company that influences whether it is more or less acceptable to engage with or accept funding from them? Some suggestions have been provided below. However, feel free to elaborate in the text box below.

	No influence	*Minor influence*	*Major influence*	*Not sure*
Size of the company (e.g. small, local company compared with large, multinational)	●	○	○	○
The healthiness of the food and/or beverages the company makes	●	○	○	○
How ethical the company is	●	○	○	○
Whether you have an existing relationship with the company	●	○	○	○
The relationship your employer has with the company	●	○	○	○
Level of trust you have in the company and individuals you will be liaising with	●	○	○	○
Whether the company operates in a sustainable manner	●	○	○	○
The size of the funding they are offering	●	○	○	○
Whether the company has expertise and capacity it is willing to share	●	○	○	○
Whether the company undertakes corporate social responsibility activities	●	○	○	○

Are there any other factors that would influence your decision-making process?

Question 3

COI (v. 1_11/04/2017)

The next question is about engaging with different types of food companies assuming you intend to follow best practice regarding conflict of interest.

Part A asks in your opinion **which commercial food and/or beverage organisations are suitable partners to accept *direct funding* from** (e.g. research grants).
Part B asks in your opinion **which commercial food and/or beverage organisations are suitable partners to accept *contributions in kind from*** (e.g. conference attendance, travel costs, assistance in printing reports etc.).
Part C asks in your opinion **which food and/or beverage organisations are suitable partners to engage with in *formal dialogue*** (e.g. exchanging information, trying to influence their internal organisation policies – no funding is involved).

NB. This is not an exhaustive list of types of commercial food organisations and some organisations will operate across multiple food sectors. It is a starting point to begin a consensus process. There is a free text box at the end of the question if you would like to add any further comments.

Food organisation	***A) direct funding e.g. research grants***			***B) accepting in kind contributions e.g. conference attendance, travel costs***			***C) engaging in formal dialogue e.g. advising on policy interventions within organisation (no funding received)***		
	Appropriate	*May be appropriate*	*Not Appropriate*	*Appropriate*	*May be appropriate*	*Not Appropriate*	*Appropriate*	*May be appropriate*	*Not Appropriate*
Food production									
Individual meat and livestock companies	◉	○	○	◉	○	○	◉	○	○
Fruit or vegetable growers	◉	○	○	◉	○	○	◉	○	○
Nut, seed or legume producers	◉	○	○	◉	○	○	◉	○	○
Egg producers	◉	○	○	◉	○	○	◉	○	○
Fish and seafood companies	◉	○	○	◉	○	○	◉	○	○
Grain growers	◉	○	○	◉	○	○	◉	○	○

Olive oil companies	◉	○	○	◉	○	○	◉	○	○
Other oil companies	◉	○	○	◉	○	○	◉	○	○
Trans-national agricultural companies (e.g. seed or food production companies)	◉	○	○	◉	○	○	◉	○	○

Question 3 (continued)

COI (v. 1_11/04/2017)

Food organisation	***A) direct funding e.g. research grants***			***B) accepting in kind contributions e.g. conference attendance, travel costs***			***C) engaging in formal dialogue e.g. advising on policy interventions within organisation (no funding received)***		
	Appropriate	*May be appropriate*	*Not Appropriate*	*Appropriate*	*May be appropriate*	*Not Appropriate*	*Appropriate*	*May be appropriate*	*Not Appropriate*
Food processing									
Dairy companies that produce a range of dairy products	○	●	○	●	○	○	●	○	○
Bread companies	○	●	○	●	○	○	●	○	○
Pasta and rice companies	○	●	○	●	○	○	●	○	○
Cereal companies	○	●	○	●	○	○	●	○	○
Cured and processed meat companies	○	●	○	●	○	○	●	○	○
Margarine companies	○	●	○	●	○	○	●	○	○
Processed fish and seafood companies (e.g. breaded fish, fish fingers)	○	●	○	●	○	○	●	○	○
Companies that produce high fat/high sugar/high salt snack products	○	●	○	○	●	○	●	○	○

Soft drink only companies	○	●	○	○	●	○	●	○	○
Bottled water only companies	○	●	○	○	●	○	●	○	○
Fruit juice only companies	○	●	○	○	●	○	●	○	○
Infant formula companies	○	●	○	○	●	○	●	○	○
Trans-national food and beverage companies with multiple ranges of products	○	●	○	○	●	○	●	○	○
Food supplement companies	○	●	○	○	●	○	●	○	○

Question 3 (continued)

COI (v. 1_11/04/2017)

Food organisation	***A) direct funding e.g. research grants***			***B) accepting in kind contributions e.g. conference attendance, travel costs***			***C) engaging in formal dialogue e.g. advising on policy interventions within organisation (no funding received)***		
	Appropriate	*May be appropriate*	*Not Appropriate*	*Appropriate*	*May be appropriate*	*Not Appropriate*	*Appropriate*	*May be appropriate*	*Not Appropriate*
Food retail/service									
Grocery retailers (e.g. supermarkets, convenience stores)	○	◉	○	○	◉	○	○	◉	○
Quick Service Restaurants (e.g. takeaway)	○	◉	○	○	◉	○	○	◉	○
Full service restaurants/cafes/pubs	○	◉	○	○	◉	○	○	◉	○
Food vending companies	○	◉	○	○	◉	○	○	◉	○

Question 3 (continued)

COI (v. 1_11/04/2017)

**Food organisation**	**A) direct funding e.g. research grants**			**B) accepting in kind contributions e.g. conference attendance, travel costs**			**C) engaging in formal dialogue e.g. advising on policy interventions within organisation (no funding received)**		
	Appropriate	May be appropriate	Not Appropriate	Appropriate	May be appropriate	Not Appropriate	Appropriate	May be appropriate	Not Appropriate
**Representative bodies (including unions)**									
Bodies that represent fish and seafood industry	○	◉	○	○	◉	○	○	◉	○
Bodies that represent meat and livestock industry	○	◉	○	○	◉	○	○	◉	○
Bodies that represent the dairy industry	○	◉	○	○	◉	○	○	◉	○
Bodies representing sugar growers and refiners	○	◉	○	○	◉	○	○	◉	○
Bodies representing bread and cereal companies	○	◉	○	○	◉	○	○	◉	○
Bodies representing manufactured food and beverage companies	○	◉	○	○	◉	○	○	◉	○
Independent agencies with	○	◉	○	○	◉	○	○	◉	○

food industry funding										

Are there any other commercial food companies/associations which you have strong feelings on whether they are suitable to accept funds from or to engage with?

Question 4

COI (v. 1_11/04/2017)

Are there any issues which have not been raised in this survey that you feel are important?

- (●) Yes
- ( ) No

Please explain

COI (v. 1_11/04/2017)

You are not eligible to participate further in this survey at the moment.

Please click Submit to exit the survey.

Thank you

COI (v. 1_11/04/2017)

Thank you for your time.

If you have any questions, please contact Katherine Cullerton on coi@mrc-epi.cam.ac.uk

Thank you

COI (v. 1_11/04/2017)

Thank you for participating in this survey.

You will be contacted again in approximately 3-4 weeks with the results of the feedback by all participants and given the opportunity to make further suggestions.

If you have any questions, please contact Katherine Cullerton on coi@mrc-epid.cam.ac.uk
